# Political Leaders’ Communication Strategies during COVID-19 in Highly Infected Countries: A Scoping Review

**DOI:** 10.3390/healthcare12060607

**Published:** 2024-03-07

**Authors:** Magde Mohamed Nour, Adnan Kisa

**Affiliations:** 1 School of Health Sciences, Kristiania University College, Prinsens gate 7-9, 0107 Oslo, Norway; adnan.kisa@kristiania.no; 2 Department of Information Science and Media Studies, University of Bergen, Fosswinckels gate 6, 5007 Bergen, Norway

**Keywords:** COVID-19, leaders’ communication, leadership, crisis communication, strategies

## Abstract

This scoping review maps communication strategies employed by political leaders in countries that experienced high infection rates during the COVID-19 pandemic. Using the Arksey and O’Malley scoping review framework, this study systematically explored the literature from 2019 to October 2023. The process involved identifying and selecting relevant studies, charting them, and summarizing the data from the 40 articles that met the inclusion criteria. This review identified a diverse array of communication strategies, which highlight the complex nature of crisis communication. These strategies featured the use of social media, science-based policy communication, strategic narrative control, empathy, ideological influences, and storytelling. These six approaches underscore the importance of adaptability and context-specific strategies in political leadership during a health crisis. The findings demonstrate that political communication during the pandemic varied significantly and was influenced by factors such as media platform, political ideology, gender, and non-verbal cues. This review enriches our understanding of crisis communication in political contexts. It emphasizes the necessity of combining traditional and digital media and considering various sociopolitical factors. The insights gained are crucial for enhancing crisis management and public trust, and they set the stage for further research and practical application in crisis communication.

## 1. Introduction

The COVID-19 pandemic tested the capabilities and limits of political leaders worldwide. It forced them to navigate uncharted territories and make lifesaving decisions under extreme pressure. In this context, as articulated by Barry (2009), “In the next pandemic, be it now or in the future, be the virus mild or virulent, the single most important weapon against the disease will be a vaccine. The second most important will be communication” [1]. Acknowledging Barry’s emphasis on communication, this study focused on mapping the literature on communication strategies employed by those leaders in highly infected countries, with a goal of understanding their effectiveness during the unexpected crisis of COVID-19.

Crisis communication involves navigating myriad factors: the nature and severity of the crisis, the cultural and socio-political milieu, and the credibility and authority of the leaders themselves [2]. These elements all influence the strategies adopted by the leaders to manage public perceptions and responses. An understanding of these strategies and their impacts is essential to identifying areas for improvement and to craft effective crisis communication techniques.

The pandemic prompted a surge in research exploring various aspects of crisis communication, including the roles and impacts of political leaders. Scholars and experts have examined the communication strategies employed by politicians in different countries, analyzing their effectiveness in terms of controlling the spread of the virus, maintaining public trust, and addressing society’s needs during the crisis. The existing literature highlights both successes and failures, providing valuable lessons to be learned.

Widely recognized in the literature is the importance of communication in promoting public understanding and compliance. Fullwood et al. (2020) emphasized that leaders who provide simple and easy-to-follow messages are more likely to increase public understanding and compliance, a view supported by other scholars [3,4]. Hu and Zhong (2023) note that effective communication not only promotes public engagement and encourages compliance, it also strengthens partnerships with the people and instills public confidence [5]. Further supporting this view, scholars found that effective responder communication, especially in the delivery of practical health-focused information, results in the highest levels of compliance [6,7]. They also stressed the need for intensified and diversified risk communication by policymakers and public health authorities to enhance public compliance and deter inappropriate behaviors during any future pandemic.

A study in 2021 reported the importance of intensified public health risk communication. Such communication should aim to increase information levels and facilitate compliance with public health interventions, including community quarantine [8]. Additionally, other researchers noted that public cooperation and compliance with restrictions received considerable attention from government and public health officials [9].

Some studies have focused on the importance of clear and consistent messaging in crisis communications. For instance, Moreland et al. (2020) argued that political leaders who spread simple and actionable messages are more likely to increase the likelihood of public understanding and compliance [10]. Another researcher emphasized the importance of empathy and emotional intelligence in communication during a crisis [11]. These studies suggest that leaders who demonstrate empathy and understanding are more likely to connect with the public at an emotional level, thereby facilitating a sense of solidarity and trust.

Additionally, the literature has revealed the importance of the media on political leaders’ communication. The government relies on media channels to get out the message. Several studies have examined the relationship between political leaders and the media, investigating their interactions, strategies, and the impact of media coverage on public perception and behavior during a crisis [12,13].

To explore the specific strategies employed by leaders, it is essential to consider the role of communication. Political leaders have adopted various strategies to manage COVID-19-related issues and communicate country-specific priorities [14]. Furthermore, the effectiveness of policy interventions depends on the credibility of political leaders and public health authorities [15].

It is also important to consider the impact of gender on the management of COVID-19. Research has examined whether having a female leader during the pandemic was associated with a lower number of COVID-19 cases and deaths per capita, after controlling for country-level cultural values [16]. Some scholars reported a connection between female leaders and the efficient performance of nations during COVID-19, providing further insights into the role of women in leadership positions during the pandemic [17].

Overall, the literature supports the notion that by delivering clear, actionable, and practical information, leaders can enhance public engagement, strengthen collaborative partnerships, and increase compliance with public health interventions and regulations [18,19,20].

Despite extensive research on leaders’ communication, there are still gaps in our understanding of the subject. The literature has provided insights into various leaders’ crisis communication strategies, but there is a lack of comprehensive understanding of these strategies, particularly in the context of COVID-19. Previous studies have focused on individual aspects such as media use or rhetorical analysis, often in isolation and within limited regional contexts. Furthermore, the nature of the crisis, with its global impact and unique challenges, requires a specific investigation of how leaders adapted their communication strategies.

This review aims to address this gap by mapping the diverse communication strategies employed by political leaders in highly affected countries during the recent pandemic. It encompasses a diverse range of cultures, personal liberties, and political systems. By doing so, this study not only contributes to the existing body of knowledge on political communication during health crises, it also provides a framework that can inform future strategies in similar emergencies, guide future research endeavors, and assist policymakers in developing effective crisis communication strategies.

## 2. Materials and Methods

### 2.1. Study Design

A scoping review methodology was chosen to thoroughly understand the existing literature on leadership communication strategies during COVID-19 in highly infected countries. This approach, which is effective for exploring new research areas and providing actionable evidence, was guided by the frameworks of Arksey and O’Malley (2005) and Levac et al. (2010) [21,22]. This method’s inclusivity allows one to consider a broad range of literature types such as primary research studies, systematic reviews, meta-analyses, letters, guidelines, websites, and blogs [23]. Our aim was to map the key concepts, sources, and types of evidence—with a focus on scholarly articles—and to identify any gaps in the research.

The selected approach emphasizes flexibility and an abductive logic of inquiry that favors a narrative-driven synthesis of findings. This approach, which is inherently interpretive, aligned well with the qualitative nature of the research topic. The review process adhered to the PRISMA protocol as outlined by Moher et al. (2015), thereby ensuring systematic and transparent reporting of findings from high-quality scientific publications [24].

The review was structured around the first five stages as recommended by Arksey and O’Malley (2005) [21] and further refined by Tricco et al. (2016) [23]. These stages are (1) identifying the research question, (2) identifying relevant studies, (3) study selection, (4) charting the data, and (5) collating, summarizing, and reporting the results. While the sixth consultation exercise stage was not conducted, it remained an available extension of this framework. Furthermore, in line with the guidance of Levac et al. (2010) [22], this review employed an interactive team approach for study selection and data extraction. It combined both a numerical summary and a qualitative thematic analysis of the extracted data, and aimed to articulate the implications of the study findings for policy and practice in crisis communication.

### 2.2. Search Strategy

Our research team consisted of two reviewers, who are the authors of this work. One is a PhD fellow with a medical degree and a master’s in public health specializing in communication, and the other is a professor with a PhD in health leadership, policy, and management. Through a combination of in-person and virtual meetings, we formulated the overarching research question and outlined the study protocol. This protocol entailed defining the search terms, identifying the databases for the literature search, establishing inclusion and exclusion criteria, and deciding on methods for resolving any disagreements among the reviewers. In October 2023, we conducted a comprehensive search based on a structured three-step strategy. Initially, a systematic search process was carried out across three major electronic databases: Medline, Embase, and Web of Science. These were selected for their extensive coverage of relevant medical and health literature. The search targeted studies published from 2019, the onset of COVID-19, to October 2023, to encompass the most relevant and contemporary studies pertaining to the pandemic. The selection of search terms was an iterative process, refined by initial search results and consultation with experts. These terms were “leadership”, “communication”, “strategy”, “COVID-19”, “SARS-CoV-2”, “pandemic”, “crisis management”, “public health” and “highly infected countries”. These were supplemented by “collaboration”, “coordination”, “decision-making”, “empathy”, “stakeholder engagement” and “communication”.

The initial database search was followed by a meticulous analysis of titles, abstracts, and index terms of the retrieved papers. Discrepancies among the reviewers were resolved through consensus meetings. Subsequently, a second search using the finalized search terms was conducted across all selected databases. Lastly, hand-searching was performed to identify any studies that may have been missed in the electronic searches. This hand-searching involved scrutinizing the reference lists of the screened studies to ensure thorough coverage and to capture any additional pertinent literature. Only peer-reviewed research articles were included, ensuring the credibility of the sources used in our analysis.

### 2.3. Selection Criteria

To be included in the review, an article had to meet the criteria listed below.

#### 2.3.1. Inclusion Criteria

- Primary studies reporting on political leaders’ communication strategies during the COVID-19 pandemic;
- Studies that focused on Argentina, Brazil, Colombia, France, Germany, India, Indonesia, Iran, Italy, Mexico, the Philippines, Poland, Romania, Russia, South Africa, Spain, Turkey, Ukraine, the United Kingdom (UK), and the United States (US). These “top 20” countries were selected based on their high rates of infection to provide a comprehensive overview across a wide array of geographic and socio-political environments, enhancing the study’s relevance and applicability in understanding the dynamics of crisis communication during a major health crisis.

#### 2.3.2. Exclusion Criteria

- Studies that did not focus on political leaders’ communication (e.g., clinical studies, vaccine studies);
- Studies focusing on lower levels of authority (e.g., organizations, hospitals, local authorities);
- Non-scholarly articles such as letters to editors, opinion pieces, monographs, book chapters, research reports, meeting abstracts, editorials, commentaries, and review articles;
- Articles not published in the English language.

### 2.4. Data Extraction

Two reviewers independently extracted data from the included studies using a predefined data extraction form. The extracted information included the first author, publication year, country of study, leadership strategy discussed, methodology, key findings, and identified areas for improvement in leadership strategy. Discrepancies were resolved through discussion and, if required, consultation with a third reviewer. A Preferred Reporting Items for Systematic Reviews and Meta-Analyses (PRISMA; Moher et al., 2015 [24]) statement flowchart was constructed to clearly outline how the included studies were selected.

### 2.5. Data Synthesis

Data were synthesized in a narrative form, guided by the objectives of the review. We grouped findings according to the identified leadership communication strategy and summarized information regarding the contexts in which the strategies were used, their impacts, and suggested areas for improvement. Tables and figures were used to categorize key aspects of the data.

## 3. Results

**Figure 1:**
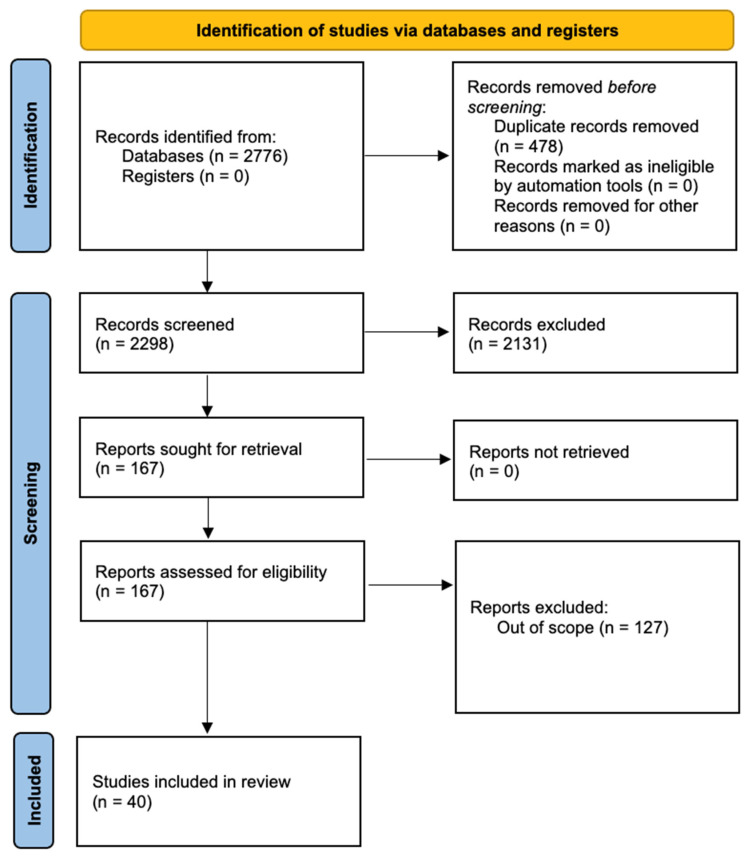
PRISMA flowchart highlighting the selection process.

Our systematic scoping review, illustrated in Figure 1, commenced with an initial search yielding 2776 records. After the removal of duplicates and the application of inclusion and exclusion criteria, 167 records were selected for full-text screening. This process yielded 40 publications that met our eligibility criteria. The selected publications, which encompass a variety of methodological approaches, provide an extensive examination of leaders’ communication strategies during the pandemic. These include quantitative content analysis, qualitative approaches, mixed methods, econometric analysis, survey analysis, and policy analysis.

The studies spanned a diverse range of countries and regions, with some countries appearing in more than one study. The United States was examined in the highest number of articles (11), constituting 21.2% of the total appearances. This was followed by the United Kingdom and Germany, with 9 (17.3%) and 7 (13.5%) appearances, respectively. Brazil, Italy, and Spain each showed up in 6 articles, accounting for 11.5% of the total for each country. At the bottom of the list were Mexico (2 articles; 3.8%) and Greece, Nigeria, India, the Philippines, and South Africa, with 1 appearance each, or 1.9%. The chronological distribution of the articles is detailed in Table A1.

This review embraces a transdisciplinary perspective to enable holistic strategies in extracting the core of various communication strategies as well as in assessing their impacts, challenges, and areas for improvement.

Based on the existing literature and guided via thematic analysis, we noted six communication strategies that could be distinguished by their unique features and objectives: (1) utilization of social media, (2) science-based policy communication, (3) strategic narrative control, (4) nonverbal communication, (5) ideologically influenced communication, and (6) metaphors and storytelling. Table A2 summarizes the strategies, areas for improvement, specific lessons learned, and opportunities for future action. Such categorization was fundamental in organizing the data to enable a coherent comparison and synthesis across the 40 articles, thereby facilitating a comprehensive understanding of these strategies within varied geopolitical contexts.

### 3.1. The Six Strategies

#### 3.1.1. Utilization of Social Media

One of the most popular communication strategies identified in our review was the use of social media. This strategy was featured in four studies conducted across various countries. Each offered unique insights into the use and impact of social media in crisis communication.

In particular, the use of social media platforms for direct public interaction and dissemination of crisis-related information was highlighted in the United States and Spain [25]. The Spanish government closely adhered to the health ministry’s recommendations on its social media profiles; it disseminated accurate and guideline-consistent information. Conversely, in the USA, while official accounts such as the White House and CDC complied with these guidelines, President Trump’s account displayed a more political bent that often contradicted public health messages. This dichotomy highlights the potential for misinformation and the influence of ideologies on communications. The study underscored the effectiveness of these platforms in engaging the public, while also noting the challenges in managing the balance between providing too much or too little information. A significant concern was the potential for misinformation. Nonetheless, the ability of social media to disseminate accurate information while countering misinformation was emphasized.

From India, a Twitter analysis examined 12,128 tweets from 29 local politicians [26]. The findings revealed that more than half of the tweets shared fact-based information and approximately 90% conveyed positive or neutral information. This study demonstrated the use of Twitter for direct communication, sentiment analysis to gauge public opinion, and dissemination of fact-based and reassuring information. However, the challenge of verifying the authenticity of follower accounts and records was noted.

A study in Italy examined the impact of government and press communication on public apprehension during the COVID-19 crisis [27]. After analyzing more than 200,000 tweets, the study found that specific types of government and media messages were positively correlated with public fear levels, particularly discussions on individual vulnerability and external control. This study emphasized the use of social media for government communication, the importance of monitoring public emotional responses through hashtag campaigns, and the necessity of quelling panic through specific messaging strategies.

A contrasting result was obtained by scholars who analyzed government Twitter communications during the first surge of COVID in Brazil [28]. This study reported that the Brazilian authorities underestimated the magnitude of the pandemic, which was reflected in their social media guidance, situational information, and even misinformation. The lack of coordination in communication among different levels of government was seen as a contributing factor in the public’s willingness (or lack thereof) to follow measures to reduce the spread of COVID.

Collectively, the studies illustrate the positive and negative impacts of social media in political leaders’ communication during the crisis. Together, the findings underscore the importance of providing accurate information and managing public sentiment during a health crisis.

#### 3.1.2. Science-Based Policy Communication

This strategy was evident across various countries, with each adopting its own approach. In the United Kingdom, the government relied on a small group of specialist advisors, particularly the Scientific Advisory Group for Emergencies (SAGE) [29]. The government used a phased response, one that initially focused on behavioral modification, and later shifted to direct regulation and lockdown. Although intended to manage the pandemic in the long term and avoid a second peak, this approach faced criticism for its slow response and failure to consider a wider range of scientific opinions. Another study looked into leaders in the UK, Italy, and the European Commission and analyzed how they utilized science in their speeches during the pandemic [14]. The researchers identified three main narratives: national pride, ethics, and integration. Each was prevalent in the communication of the different leaders. The study highlighted the use of science to build national identity and pride, emphasized social responsibility, and underscored the role of science in the economy, social development, and political identity.

A study from the Philippines examined the government’s policy responses and the role of scientific advice in managing the pandemic [30]. The study stressed the importance of scientific advice in shaping policy decisions and underlined the challenges in implementing effective measures during the pandemic. In South Africa, a study analyzed President Ramaphosa’s communication approaches during the crisis [31]. Unlike other presidents, Ramaphosa relied on scientific advice and a diplomatic approach, which bolstered public trust. However, the study pointed out the challenges in this approach, including potential elitism and little community input, which may have contributed to a lack of compliance with public health measures.

These studies illustrate the importance of relying on scientific advice for policy communication. They also draw attention to the challenges in managing public perception, ensuring inclusivity, and addressing the socioeconomic realities of different populations.

#### 3.1.3. Strategic Narrative Control

One striking communication strategy was noted in Spain, Italy, Germany, and Mexico [27,32,33]. The Spanish government’s communications can be described as a blend of conventional information and a subtle use of warlike language. This approach featured a narrative that played up the pandemic’s severity. Such a strategy appeared to be a deliberate choice to impose social control and compliance through a somewhat alarmist narrative, aligning with broader crisis management efforts.

The study from Italy looked into how government and press releases affected public anxiety [33]. The authors emphasized the significance of using empathetic and targeted messaging strategies to address public concerns.

The Mexican government disseminated information primarily through press conferences. The article described a blend of rational and emotional messaging while also pointing out the difficulty in balancing technical content with relatable, engaging communication [27].

Collectively, these studies explain the application of narrative control and public engagement in governmental communication during COVID. They emphasize the importance of empathetic communication and the effectiveness of both traditional and digital communication channels. Challenges in this strategy include aligning communications with public needs and perceptions. There is, therefore, a need for a wide variety of communication approaches to manage public perception and response during a health crisis.

#### 3.1.4. Nonverbal Communication

This strategy was often employed in Spain and several other Western democracies. In Spain, the televised speeches of President Pedro Sánchez revealed a notable control in his body language and vocal expressions coupled with excessive repetition in body movements [34]. There was a significant disconnect between his verbal communication and nonverbal cues (both kinesic and paralinguistic), which undermined the credibility of his words. The study highlights a strategic but inconsistent integration of nonverbal elements with his verbal messages, especially across different phases of the pandemic.

Another pivotal study, “Gender Effect on Political Leaders’ Nonverbal Communicative Structure during the COVID-19 Crisis”, analyzed the televised appearances of 10 heads of state (five males and five females) from various democratic Western countries. Four of these countries—the US, UK, Italy, and Germany—were included in this review’s scope [35]. The study revealed significant differences in nonverbal communication structures between men and women. Men displayed more assertive, controlling, and rational nonverbal behaviors, whereas women tended to show empathetic, cooperative, and emotionally communicative nonverbal cues. Interestingly, according to the study, countries led by women had fewer severe COVID-19 cases, suggesting a possible link between leadership styles and pandemic outcomes. The study raised concerns about the risk of reinforcing gender stereotypes through nonverbal communicative structure and how gendered communication might influence public perception and crisis management effectiveness differently.

In summary, these studies determined the critical role of nonverbal communication and the implications of this strategy in public perception, crisis management, and health outcomes. These findings reinforce the importance of considering verbal and nonverbal communication in evaluating political leadership during a health crisis.

#### 3.1.5. Ideologically Influenced Communication

Numerous studies have underscored ideologically influenced communication as a key strategy among political leaders during the pandemic, with a focus on South America, particularly Brazil, alongside notable instances in the United States and Mexico [36,37,38,39,40]. This body of research, particularly those articles covering Brazil, has been pivotal in revealing the influence of ideologies on public communication in times of crisis.

Significant among these is a work that explored Brazilian President Bolsonaro’s communications [37]. The author found a distinctive “populist-crisis” mode characterized by messages about the people and opposing elites, leading to further social divisions and misinformation.

A study by Stuart Davis and co-authors (2023) [39] linked President Bolsonaro’s response to the pandemic with elements of right-wing populism and epidemiological denialism, leading to public mistrust in health institutions. Similarly, a critique by Elize Massard da Fonseca and colleagues (2021) [40] examined Bolsonaro’s denialism and prioritization of economic interests over health considerations, which led to tensions between various levels of government. Damasio Duval Rodrigues Neto (2021) [41] also critiqued the Brazilian government’s response, particularly its handling of the pandemic and the promotion of free market legislation.

In the United States, scholars revealed patterns of “follow the leader” politics and “responsive representation” [38]. In Mexico, Erika Lourdes González-Rosas and her co-authors (2022) [42] delved into President López Obrador’s utilization of Twitter to reveal a lack of effective communication regarding the health emergency.

These studies all demonstrate the role that politics played in shaping communication during the COVID-19 pandemic. The ideologies of the leaders profoundly influenced their messages, leading to public mistrust, crisis mismanagement, and the potential for misinformation. These insights highlight the dangers of mixing politics with public health.

#### 3.1.6. Metaphors and Storytelling

The employment of metaphors and storytelling during the pandemic forms the crux of a comprehensive study conducted in Greece [43]. Gkalitsiou and Kotsopoulos meticulously documented how metaphors and stories were not just rhetorical flourishes but became propaganda tools that were increasingly utilized as the crisis intensified. The authors categorized the types of metaphors and narratives employed, shedding light on their use to bolster communication effectiveness, convey pivotal messages, and influence public perception.

The study found some inherent challenges of this approach. A significant concern is the propensity for metaphors and stories to simplify complex realities. The study also draws a line between rhetorical efficacy and factual integrity, highlighting instances where metaphorical language could obscure or distort factual content. While acknowledging a story’s capacity to engage and sway audiences, the study cautions against their indiscriminate use. This research contributes significantly to the discourse on political communication, particularly in crisis contexts. It emphasizes the importance of audience interpretation and the specificities of the crisis in determining the impact of such rhetorical strategies.

## 4. Discussion

This scoping review offers an extensive mapping of the communication strategies deployed by political leaders during the COVID-19 pandemic in the highly infected countries, highlighting both congruence with and divergence from established theories in political crisis communication. Notably, the strategic employment of social media in nations such as the USA, Spain, India, and Brazil aligns with Chadwick’s hybrid media system theory, which emphasizes the integration of traditional and digital platforms in political discourse [44]. However, challenges like misinformation and the sway of political ideology on communication methods mark a significant departure from traditional models, which often regard social media as straightforward tools for factual dissemination.

Further, the examples of the UK and South Africa resonate with Bucchi’s model of public engagement in science communication, underscoring the significance of scientific input in policymaking [45]. And yet, complaints of slow responses and potential elitism in these approaches suggest a more intricate interaction between science and policy than often presumed.

Our analysis also uncovered cases of strategic narrative control, empathy, and public engagement in such nations as Germany and Mexico, reflecting Entman’s framing theory [46]. This strategic narrative shaping, along with traditional communication methods and empathetic language, illustrates a complex dynamic in crisis communication, challenging existing models that often overlook the intricacies of empathetic and authoritative communication across varied cultural contexts.

Our review also explored the role of nonverbal communication and gender differences, especially in Western democracies. By doing so, our review supplements theories on nonverbal cues in political discourse and challenges gender stereotypes in leadership, as posited by Ekman [47] and Eagly and Carli [48]. This calls for a more inclusive approach to gender dynamics in political communication.

The influence of ideologies on strategic communication in, inter alia, South America and the USA aligns with Corner and Pels’ concept of political communication as an ideological tool [49]. Its impact on public trust and crisis management echoes Bennett and Iyengar’s concerns about polarization in crisis communication, challenging views of strategic communication as a neutral conduit [50].

The use of metaphors and storytelling, particularly in Greece, bears out Lakoff and Johnson’s metaphor theory in politics, highlighting the challenges of maintaining rhetorical effectiveness without compromising factual accuracy [51]. This necessitates the use of refined theories that address the pitfalls of employing metaphors in crisis communication.

The review also identifies unexpected and occasionally contradictory findings, such as the effectiveness of traditional communication methods versus the growing reliance on digital platforms. For instance, Angela Merkel’s use of direct and clear information through televised speeches offers a stark contrast to the prevalent advocacy of social media in crisis communication. This suggests the context-dependent efficacy of communication strategies, influenced by cultural norms, public expectations, and the nature of the crisis.

Additionally, this review highlights the significance of gender on nonverbal communication strategies, challenging traditional notions in political communication literature. This finding suggests a possible link between communication style and crisis outcomes, necessitating cautious interpretation to avoid reinforcing gender stereotypes.

Contradictory findings in the utilization of social media illustrate the complex landscape of digital communication in crises, where benefits such as reach and immediacy are offset by risks of misinformation and political manipulation. This underscores the need for research to develop balanced strategies that capitalize on social media’s strengths while mitigating its drawbacks.

While this study focused on political leaders, it is important to recognize the influence of religious leaders on public discourse. This aspect, extensively explored by Norberto Gonzalez Gaitano [52], highlights the diverse sources of leadership and their communication strategies, adding an important dimension to the broader narrative of leadership during public health emergencies.

The practical implications of these findings are useful for political leaders and communication strategists. An effective approach to crisis communication requires a strategy that seamlessly integrates traditional and digital platforms, is tailored to specific cultural and political contexts, and considers factors such as gender and nonverbal cues. This underscores the importance of developing adaptive communication strategies that can respond to the changing needs of diverse audiences and evolving circumstances.

Equipping political leaders with a toolbox of communication skills, including mastery of nonverbal cues and empathetic language, could enhance their ability to connect with and reassure the public. Moreover, establishing robust frameworks to counteract misinformation, coupled with efforts to promote media literacy among the public, are essential steps in augmenting the efficacy of crisis communication. Such strategies not only empower leaders in conveying their messages more effectively, they also enable the public to evaluate and respond to the information they are bombarded with. This nuanced approach is pivotal in navigating the complexities of modern political discourse and ensuring that public messaging during health crises is both trustworthy and useful.

Future research should explore the adaptability of communication strategies across different cultural and political sceneries and in response to the turbulent media and technological landscape. The digital era has ushered in transformative changes in communication, presenting both opportunities and pitfalls for leaders. Understanding how to tailor communication strategies to diverse contexts will further enrich our mastery of crisis communication. Additionally, future studies should explore how leaders can balance the dissemination of accurate, science-based information with real audience engagement, especially on digital platforms. The rise of social media has revolutionized the way leaders communicate during crises, but the challenge remains to maintain informational integrity while ensuring robust audience engagement.

Moreover, there is a need to move beyond stereotypes to understand how gender dynamics influence the efficacy of crisis communication.

Longitudinal studies are also necessary to evaluate the long-term effects of various communication strategies on public trust, policy compliance, and crisis resolution. Understanding these long-term impacts will guide policymakers and practitioners in choosing the most effective approaches to crisis communication.

## 5. Conclusions

This scoping review has systematically outlined the variety of communication strategies utilized by political leaders during the COVID-19 crisis. It uncovered the nuanced interplay of social media, science-based policy communication, narrative control, empathy, public engagement, and both traditional and digital platforms. This analysis not only deepens our understanding of health crisis communication in a political context but also reveals the strengths and limitations inherent in these diverse approaches.

A takeaway from this study is that giving clear, calm, and honest messages in the midst of a deadly pandemic is no easy job. The varied strategies adopted by leaders around the globe affirm that effective communication in times of crisis cannot be restricted to a single methodology. Rather, it necessitates a bespoke approach, finely tuned to the distinct cultural, societal, and political fabrics of each audience. This insight calls for political leaders to be aware of their audience’s cultural sensitivities, values, and preferences when crafting their communication strategies.

The influence of ideologies on communication, particularly in regions like South America, underscores the intertwining of political bias with public trust and the efficacy of crisis management. Leaders need to transcend their biases and ensure that their communication strategies align with the broader interests of the public.

Furthermore, this study highlights the role of gender differences in nonverbal political messaging. These differences call for a sophisticated approach to engaging with diverse audiences, enhancing the leaders’ capacity to project empathy, establish trust, and effectively convey their messages during crises.

Additionally, the enduring relevance of traditional media, exemplified by Angela Merkel’s televised addresses in Germany, shows that, notwithstanding the popularity of social media, traditional channels remain vital in reaching broad audiences and maintaining credibility. Leaders are thus encouraged to leverage a multi-platform approach that capitalizes on the unique strengths of different communication channels.

This review also recognizes its own limitations. Its focus on leaders’ strategies and the selection of English language publications potentially neglects the broader spectrum of crisis communication stakeholders and non-English research. Moreover, the evolving nature of the COVID-19 pandemic suggests that more recent developments in crisis communication may have been missed. The diverse methodologies of the analyzed studies also introduced variances in quality and depth, impacting the uniformity and robustness of the conclusions drawn. Furthermore, the search conducted on Web of Science, Embase, and Medline, though extensive, did not encompass the entirety of available literature. While these databases offer a broad coverage of peer-reviewed journals and have an international reach, they do not include all available evidence.

## Data Availability

The data presented in this study are available upon reasonable request from the authors of this manuscript.

## Appendix A

**Table A1:** Characteristics of included studies with location, study type, key findings, communication strategies, and challenges.

Author(s) and Year	Location	Study Type	Key Findings	Communication Strategies	Challenges and Limitations
Watkins and Clevenger, 2021 [53]	USA	Political discourse analysis	US leaders exhibited diverse political vulnerabilities linked to crisis management, with perceptions shaped by leadership style, behavior, and contextual factors.	Transparency and adherence to scientific evidence in briefings.	Difficulties in measuring the impact of communication strategies on public policy and health outcomes.
Nicasio Varea et al., 2023 [25]	USA and Spain	Comparative analysis	Spanish government closely adhered to health crisis communication guidelines, whereas in the USA, only White House and CDC accounts complied, with President Trump’s account displaying a politicized approach divergent from public health messages.	In Spain, leaders adhered to health crisis communication guidelines and utilized social media for direct interaction with citizens. In the USA, a politicized communication strategy was observed, often diverging from public health messages.	Challenge in balancing political agendas with effective crisis communication, the potential for social media to spread misinformation, and the influence of political ideologies on communication strategies.
Álvarez-Nobell et al., 2022 [36]	Argentina, Brazil	Comparative case study	Ideological orientations of Argentina’s and Brazil’s governments significantly impacted their public communication strategies, crisis management during COVID-19, public trust, and the effectiveness of communication channels.	Political ideologies shaped communication strategies.	Limited transparency and inconsistent messaging affected the public’s trust in government sources and the effectiveness of communication channels.
Burni, 2021 [37]	Brazil	Textual analysis	President Bolsonaro’s “populist-crisis” communication approach during COVID-19 emphasized economic challenges, centered on a people-focused narrative, and positioned him against the elites.	Populist crisis communication model.	May foster social divisions, politicize crises, and lead to misinformation and reliance on anti-science rhetoric.
Cairney, 2021 [29]	United Kingdom	Qualitative analysis	UK’s COVID-19 response led by a select group of scientific advisors, prioritizing long-term management, a phased approach transitioning from behavioral modification to regulation and lockdown.	Communicated based on specialist scientific advice.	Delayed response and narrow consideration of scientific opinions led to debates about the timing and substance of interventions, and dissatisfaction among external experts and peripheral insiders.
Cairney and Wellstead, 2021 [54]	UK and USA	Qualitative analysis	Trust in experts, politicians, and the public shaped governmental responses and the acceptance of scientific advice, influencing the effectiveness of policymaking.	Trust in scientific evidence and expert advice for policy formation, and communicating clear, honest, and respectful messages to maintain trust.	Challenges included managing the balance between trust-based and coercive measures, ensuring consistent and coherent messages, and dealing with the variable levels of trust across different political systems and societal norms.
Casalegno et al., 2021 [55]	Italy	Institutional communication analysis	Over- and under-communication exacerbated the knowledge-behavior gap, resulting in misbehaviors and incoherent responses.	Timely and synchronous communication based on interaction, participation, and relational messages.	Difficulties in striking a balance between providing adequate and excessive information, preventing misinterpretations, and tailoring communication strategies to various audience segments.
Castillo-Esparcia et al., 2020 [32]	Spain	Communication analysis	Media saturation, narrative control with war-themed rhetoric, and an economic focus in international press contrasted with public interests.	Warlike language, collaboration with specialists in decision-making, and strategic information dissemination through diverse channels.	Managing the overabundance of information, aligning government communication with public interest, and balancing clear, authoritative communication with effective social media engagement.
Chang, 2022 [56]	Taiwan and USA	Survey analysis	Taiwan’s direct government communication enhanced perceived empowerment and preventive behaviors, whereas the USA experienced lower empowerment and preventive actions due to less effective communication.	Timely, useful, and trustworthy information dissemination.	Delayed initiation of regular press conferences and less effective content in communication resulted in lower perceived government empowerment and less engagement in preventive behaviors.
Cormack and Meidlinger, 2022 [38]	USA	Analytical study	US legislators increased communications in response to local fatalities, with some echoing Trump’s terminology and advocating hydroxychloroquine, reflecting “follow the leader” politics.	Responsive representation and “follow the leader” politics, where some legislators echoed the rhetoric of the President.	Striking balance between conveying accurate public health information and navigating political narratives and partisanship may risk misleading or harming public understanding.
Crespo-Martínez et al., 2022 [57]	Madrid, Spain	Survey and binary logistic regression analysis	Agreement with “influenzaization” discourse correlated with lower risk perception and diminished support for restrictive measures, influenced significantly by ideological leanings.	Promoting the “influenzaization” discourse to downplay COVID-19 severity and employing diverse communication channels to influence public perception and attitudes.	Potential decrease in public adherence to health measures and making it difficult to maintain a balanced and responsible communication approach.
Davis et al., 2023 [39]	Brazil	Qualitative textual analysis of Bolsonaro’s public interviews, campaign speeches, and social media communication	Bolsonaro’s COVID response, rooted in right-wing populism and epidemiological denialism, eroded public trust in health institutions and vaccination, fostered blame on outsiders, and spread misinformation.	Utilizing social media for public communication. Anti-elitism and anti-pluralism communication.	Challenge in combatting public health misinformation propagated by political populism and difficulty in reinstating public trust in health institutions.
Drescher et al., 2021 [58]	Germany	Quantitative contentanalysis	Tweet frequency on COVID-19 by authorities and experts rose over time, with experts’ tweets receiving more retweets and likes, while hashtags in authority tweets diminished spread, and tweets about severity and social consequences garnered more retweets.	Use of hashtags, images, URLs, and mentions, with a focus on conveying severity, susceptibility, efficacy, and technical, social, and political consequences.	Tweets containing hashtags, structural elements, and political consequences content had lower retweet rates compared to experts’ tweets.
Drylie-Carey et al., 2020 [59]	Europe (UK, France, Spain, Italy, EU)	Quantitative contentanalysis	Leaders exhibited inconsistent transparency and coherence in Twitter communications, with notable gaps between tweets and actual policies or values, and a lack of platform-specific tailoring in their use of audiovisual content.	Utilized personal videos, selfies, and amateur material for direct communication, alongside institutional pictures and press conference videos, while actively engaging with their audience through comments, shares, and likes on social media.	Underutilized Twitter’s dialogic potential, and difficulty aligning audio-visual discourse with impactful content.
Fernández-Hoya and Zapatero, 2022 [34]	Spain	Quantitative systematization and qualitative interpretation of nonverbal communication	President Sánchez showed consistent body tension and vocal qualities, varying gestures and expressions with the contagion curve, and asynchronous intersystemic communication affecting speech credibility.	Nonverbal cues like body movements and tone of voice to reinforce verbal messages.	Inconsistent verbal and nonverbal communication, repetitive gestures, and lack of communicative coherence across pandemic phases may have affected the effectiveness of political leaders’ messaging.
Fonseca et al., 2021 [40]	Brazil	Qualitative andquantitative analysis	Bolsonaro’s emphasis on economic aspects and minimization of COVID-19 severity, coupled with the spread of misinformation and dismissal of scientific guidance, exacerbated tensions between governmental levels and impaired Brazil’s pandemic response.	The use of political rhetoric and dissemination of misinformation and pseudoscience.	The denialist approach and misinformation led to public confusion, hindered a unified response, and contributed to severe COVID-19 outbreak.
Gasulla et al., 2023 [60]	USA	Econometric analysis	Political affiliation of state leadership influenced response speed and stringency, with Democrat-led states acting more swiftly and strictly.	Communication strategies influenced by party alignment and federal–state dynamics.	Political polarization and weak executive federalism hindered a unified national response, leading to varied state-level responses and differing pandemic management outcomes across the USA.
Geurts et al., 2023 [61]	Germany, Guinea, Nigeria, and Singapore	Qualitative document review and key informant interviews	Each country exhibited unique emergency risk communication (ERC) strategies, with early integration into plans being vital; however, improvements were needed in interactive communication, community involvement, and monitoring. Countries with past pandemic experience showed better preparedness for ERC execution.	Utilization of diverse communication channels and modes, and a focus on message clarity, consistency, and relevance.	Difficulties in maintaining two-way communication and effective community engagement, challenges in monitoring, evaluation, and message adaptation.
Gkalitsiou and Kotsopoulos, 2023 [43]	Greece	Qualitative and quantitative analysis	Leaders extensively utilized metaphors and storytelling, increasing in intensity with crisis severity, and exhibited specific patterns in their metaphor and story choices.	Strategic use of metaphors and storytelling to enhance communication.	The effectiveness of metaphors and storytelling is context-dependent and can lead to misinterpretations or oversimplifications, requiring careful balancing of rhetorical impact with factual accuracy.
Gollust et al., 2020 [62]	USA	Descriptive analysis	COVID-19′s politicization resulted in varied public responses and behaviors linked to political affiliations, with partisan reasoning shaping threat perception and health actions, further intensified by differing media coverage.	Use of politically charged rhetoric and framing. Leveraging media outlets to disseminate specific narratives and information.	Politicization resulted in fragmented public responses and undermined cohesive public health messaging.
González-Rosas et al., 2022 [42]	Mexico	Qualitative analysis	López Obrador’s communication during COVID-19 combined an informative style with populist elements but lacked effective use of Twitter for emergency information dissemination, suggesting a need for adapting his style to pandemic circumstances.	Communication populism, charisma, and strategic behavior, as well as informative communication.	Ineffective use of Twitter for direct communication, limited engagement in strategic or empathetic discourse related to the pandemic, and potential misalignment between communication style and the situation’s needs.
Grebelsky-Lichtman et al., 2020 [35]	USA, UK, Israel, Italy, Canada, Belgium, Finland, Denmark, New Zealand, and Germany	Mixed-method approach	Gender differences in national leaders’ nonverbal communication structures (NCS) during televised appearances correlated with COVID-19 outcomes: male leaders displayed competitive and aggressive behaviors, while female leaders demonstrated cooperative, empathetic, and optimistic communication, associated with better health outcomes.	Emotional and empathy communication.	Potential risk of reinforcing gender stereotypes and gendered communication may influence public perception and effectiveness differently during a crisis.
Guibarra and Sánchez, 2020 [33]	Mexico	Content analysis	Press conferences centered on government management with a blend of rational and emotional messaging, prioritizing clarity in communications.	Regular updates and information dissemination with an integration of rational and emotional messaging.	Potential for overemphasis on government actions, challenges in balancing technical information and relatability, and difficulties in maintaining message consistency and countering misinformation.
Gumede et al., 2022 [31]	South Africa	Comparative analysis	President’s distinct communication strategies, ranging from AIDS denialism to scientific approaches during COVID-19, significantly influenced public health outcomes and societal responses.	Science-based and formal communication.	Top-down communication strategy was perceived as elitist and disconnected from community realities, and had limitations in building trust and engaging with the public.
Haan et al., 2022 [63]	Germany	Quantitative analysis	Angela Merkel’s announcements extended public expectations of COVID-19 restriction durations, particularly influencing those with higher initial optimism.	Televised press conferences to disseminate policy information and maintain public vigilance.	Balancing communication to manage public expectations while avoiding panic proved challenging, as public expectations shifted in response to specific communication and policy announcements.
Hu and Zhong, 2023 [5]	USA	Quantitative analysis	Governors’ reputation concerns and politicization influenced their communication strategies, affecting public engagement and compliance, and potentially diminishing communication legitimacy and effectiveness.	Politics-oriented communication strategies; avoiding blame and protecting reputation.	Politicization hindered public engagement and compliance with policy guidance, raising concerns about the long-term impact on government legitimacy and effectiveness.
Jäckle et al., 2023 [64]	Germany	Multi-level regression analysis	Political trust fostered anti-pandemic measure acceptance, while social trust and liberal ideology increased skepticism, especially among right-leaning individuals with high social trust.	Leveraging social trust to manage community response to the pandemic.	Challenges in aligning public trust with policy acceptance across diverse ideological groups, as well as in managing the contrasting influences of political and social trust on public adherence to measures.
Kaur et al., 2021 [26]	India	Qualitative analysis	Over half of the 12,128 tweets from 29 Indian political leaders contained fact-based information, and approximately 90% were positive or neutral in tone.	Use of Twitter for direct communication with the public, sentiment analysis to assess public opinion, and dissemination of factual and reassuring information.	The authenticity of follower accounts is difficult to determine, posing challenges for evaluating the effectiveness of communication strategies.
Kneuer and Wallaschek, 2022 [65]	Germany	Qualitative contentanalysis	Angela Merkel utilized solidarity and diverse communication styles in speeches, press conferences, and weekly podcasts during the initial phase of the pandemic.	Emphasized solidarity and trust, adapting communication to different platforms and appealing for collective effort.	
Koch and Durodié, 2022 [66]	UK	Qualitative analysis	COVID-19 highlighted tensions between scientific expertise and political decision-making, underscoring difficulties in aligning expert advice with political accountability, transparency, and democratic principles.	Reliance on scientific advice and expert-led recommendations while attempting to depoliticize debates using scientific legitimacy.	Potential for scientific advice to be misused as a tool for political legitimacy, risk of undermining democratic processes, and challenges in maintaining a balance between expert advice and political decision-making.
Lawson and Lugo-Ocando, 2022 [67]	UK	Mixed-methods approach	Politicians communicated vague numerical information about COVID-19, while the media provided specific statistics, leading to a misalignment in public perception.	Selective presentation of data to shape public understanding.	Challenge of achieving coherent and consistent communication between government, media, and public, and the potential for media narratives to diverge from political messaging, impacting public perception and response.
Lerouge et al., 2023 [27]	Italy	Mixed-methodsapproach	Government and media messages about individual vulnerability and external control increased public fear in Italy.	Utilized social media to engage with the public, monitor public sentiment through hashtag campaigns, and addressed public fears with targeted messaging.	The authenticity of follower accounts is difficult to verify, posing challenges for evaluating the effectiveness of communication strategies.
Loner et al., 2023 [14]	UK, Italy, European Commission	Mixedmethods	Leaders employed national pride, ethics, and integration narratives in framing and utilizing science in their speeches.	Harnessed the power of science to promote national pride, emphasize social responsibility, and underscore the role of science in societal development and political identity.	
Mintrom and O’Connor, 2020 [68]	USA (California, Florida, New York, Texas)	Qualitative analysis	Policy narratives shaped state governments’ responses, with varied narrative strategies among state governors emphasizing the importance of narrative management in crisis situations.	Persuasive accounts of crisis events, creation of broad coalitions of support, fostering trust and cooperation, enabling informed decisions by communities, managing multiple agendas.	Challenges in aligning narratives with actions, crafting messages suitable for local contexts, and managing multiple agendas while maintaining a coherent and persuasive narrative.
Duval and Neto, 2020 [41]	Brazil	Analysis of government policies and discourses	Government inadequately responded to the pandemic, exploiting the crisis to advance pro-market legislation.	Political narratives and discourses to control public perception and policy measures.	Lack of coordination and social participation in policymaking.
Neves et al., 2022 [28]	Brazil	Analysis of Twitter communications	Government authorities underestimated the pandemic’s magnitude, leading to misinformation and a lack of coordinated response.	Using Twitter for communication, including guidance and situational information.	The lack of coordination and inconsistency in messaging across agencies and levels of government may have contributed to the public’s unwillingness to follow anti-COVID measures.
Reich, 2020 [69]	Japan and USA	Comparative assessment	Japan’s success in managing the pandemic vs. the USA is attributed to differences in governance, mask-wearing culture, social values, national leadership roles, and information clarity.	Emphasis on individual liberty, decentralized decision-making, and mixed messaging from leadership.	Challenges in changing public behavior and perceptions.
Rivas-de-Roca et al., 2021 [70]	Germany, Spain, Portugal, UK	Comparative analysis	Governments’ Twitter and website communications during COVID’s second wave varied in themes, objectives, and citizen engagement, reflecting diverse communication styles.	Neutral, institutional, emotive, and personal tone.	Disconnect between government communication objectives and public interest.
Schnabel et al., 2023 [71]	Germany, Italy, UK	Comparative analysis	Highlighted the role of political leadership and coordination in crisis response in multilevel governance systems.	Different levels of coordination and consistency among leaders, with minor differences in timing of measures across countries.	Challenges of maintaining consistent crisis communication in multilevel governance systems, with differences in political structures, leadership styles, and regional autonomy.
Vallejo Jr. and Ong, 2020 [30]	Philippines	Policy analysis	The importance of scientific advice in shaping policy decisions in the government’s response.	Combination of scientific advice and policy decisions.	Complexity and uncertainty of pandemic governance, emphasizing the need for stable and effective scientific advisory structures for crisis management.

## Appendix B

**Table A2:** Summary of communication strategies, associated positive outcomes, challenges, and areas for improvement.

Communication Strategy	Location	Description	Positive Outcome	Challenges	Areas for Improvement
1. Utilization of social media [25,26,27,28]	USA, Spain, India, Italy, Brazil	Use of social media platforms for direct public interaction, dissemination of crisis-related information, and sentiment analysis. Involved monitoring emotional responses and providing accurate information.	Effective public engagement and information dissemination.	Potential for misinformation, balance between too much/too little information, verifying follower account authenticity.	Improve information authenticity, manage misinformation, align with public interests.
2. Science-based policy communication [29,30,31,66]	UK, Italy, Philippines, South Africa	Reliance on scientific expertise and advisors in policymaking. Emphasizes the role of science in shaping policy decisions and public messages. Involves integrating scientific advice into national narratives and policy responses.	Shaping policy decisions based on scientific advice, building national identity and pride through science.	Slow response to emerging scientific opinions, challenges in implementing effective measures, potential elitism.	Broaden the range of scientific opinions considered, enhance public engagement and inclusivity.
3. Strategic narrative control, empathy, and public engagement [27,28,32]	Spain, Italy, Mexico	Regular press conferences, empathetic language, collaboration with experts, and diversified dissemination channels. Focused on clear and direct communication, often through traditional methods like televised speeches.	Reduction in anxiety and depression, effective public communication.	Information overload, difficulty aligning government communication with public interests, balancing technical content with engaging communication.	Improve alignment of communication with public interests, enhance engagement on digital platforms.
4. Nonverbal communication [34,35]	Spain, various democratic western countries	Analysis of nonverbal cues like body language, kinesic language, and paralinguistic elements. Gender differences in nonverbal communicative structure (NCS) in political leadership.	Enhanced credibility and persuasive power of speeches, potential impact on pandemic outcomes.	Lack of communicative coherence between verbal and nonverbal elements, reinforcing gender stereotypes.	Integrate of nonverbal cues with verbal communication, avoid reinforcing gender stereotypes.
5. Strategic communication and ideological influences [36,38,39,40,42]	South America (Brazil, Argentina), USA, Mexico	Communication strategies shaped by political ideologies. Involved populist-crisis communication, epidemiological denialism, and “follow the leader” politics. Emphasized people-centric messages and opposing elites.	Maintaining support base, constructing persuasive political narratives.	Fosters social divisions and misinformation, undermines public trust in health institutions.	Align communication strategies with public health needs, avoid politicization of health crises.
6. Metaphors and storytelling [43]	Greece	Employed metaphors and storytelling to convey critical messages and persuade audiences, particularly during crises. Involved strategic use of rhetorical devices to enhance communication.	Engaging and persuading the audience, enhancing communication effectiveness.	Potential for misinterpretation or oversimplification of complex issues, ambiguity in messaging.	Carefully consider rhetorical tools to maintain clarity and accuracy, context-dependent usage.
